# The Effects of a Cyberbullying Intervention Programme Among Primary School Students

**DOI:** 10.1007/s10566-022-09714-9

**Published:** 2022-10-02

**Authors:** Ágnes Lukács J., Johanna Takács, Zsuzsanna Soósné Kiss, Máté Kapitány-Fövény, András Falus, Helga Judit Feith

**Affiliations:** 1 Department of Social Sciences, Faculty of Health Sciences, Semmelweis University, Vas utca 17, Budapest, 1088 Hungary; 2 Department of Health Sciences, Faculty of Health and Sport Sciences, Széchenyi University, Szent Imre út 26-28, Gyor, 9024 Hungary; 3 Department of Addictology, Faculty of Health Sciences, Semmelweis University, Vas utca 17, Budapest, 1088 Hungary; 4 Nyírő Gyula National Institute of Psychiatry and Addictions, Lehel utca 59, Budapest, 1135 Hungary; 5 Department of Genetics, Cell- and Immunobiology, Faculty of Medicine, Semmelweis University, Üllői út 26, Budapest, 1085 Hungary; 6 EDUVITAL Foundation, Nagyvárad tér 4, Budapest, 1089 Hungary

**Keywords:** Cyberbullying, Cyberbullying involvement, Peer education, Effect, Primary school-aged students

## Abstract

**Background:**

With the increase of cyberbullying, several intervention programmes have been created that aim at reducing cyber-victimisation and perpetration.

**Objective:**

Our study presents the effects of the STAnD anti-cyberbullying programme with peer-education both on the short and the long run among lower and upper primary school students, with a focus on the participants’ cyberbullying roles.

**Method:**

The sample comprised of 536 students who participated in the intervention programme, involving 36% lower and 64% upper primary school students. Participants were measured by a self-reported questionnaire before and right after the programme, then six months later.

**Results:**

The main effect of the STAnD programme was a positive change in the participants’ willingness to engage in help-seeking and their active-defending reaction, although this effect decreased after six months. The changes were larger among lower primary school students compared to upper primary school participants.

**Conclusion:**

Our results imply that long-lasting and intensive health promotion programmes are necessary to reach a long-term intervention effect. Anti-cyberbullying programmes should take into consideration participants’ involvement and roles in cyberbullying. As our study was a non-randomised uncontrolled study design, thus interpretation of the effectiveness of the programme is limited.

**Supplementary Information:**

The online version contains supplementary material available at 10.1007/s10566-022-09714-9.

## Introduction

With the increase of cyberbullying,^1^ several intervention programmes have been created that aim at reducing cyber-victimisation and perpetration. Systematic reviews (Hutson et al., 2018; Gaffney et al., 2019; Lan et al., 2022; Ng et al., 2022; Polanin et al., 2022) summarise the characteristics of cyberbullying interventions as the followings. Interventions are generally realised in school settings and show great variety in terms of length and frequency—from one-time sessions to whole-year programmes. As for the form of these interventions, we can find lectures, group discussions, drama games, and project works. The foci of the prevention education sessions are teaching about cyberbullying, learning coping strategies, improving children’s empathy, communicative and social skills, and often the topic of internet security is included. Although there are some anti-bullying programmes with cyberbullying elements concentrated on lower primary school students (Villarejo-Carballido et al., 2019; Williford et al., 2012), anti-cyberbullying programmes target mainly upper primary and secondary school age-groups (Cantone et al., 2015). However, lower primary school-aged children’s access to technology and social media is growing and so does their involvement in cyberbullying (Ey et al., 2015).

The objective of anti-cyberbullying programmes can differ by the degree of involvement in cyberbullying, as participants could have diverse experience about cyberbullying (Del Rey et al., 2016). Furthermore, programmes should encourage the willingness of help-seeking (either professional or non-professional help), as victims rarely ask for help in a cyberbullying incident. According to the current body of scientific research, this has several explanations. Firstly, victims feel ashamed; secondly, they are afraid of the consequences (e.g., in the case of children parents limiting their internet access); thirdly, they assume that adults can do nothing against cyberbullying (Dooley et al., 2010; Smith et al., 2008; Spears et al., 2015). Since victims usually employ avoidant or depressive coping strategies, encouraging their willingness to ask for help can modify their perception about the unchangeability of the cyberbullying situation, and reduce the mental costs of victimisation (Smith et al., 2008; Völlink et al., 2013). Regarding bullies, increasing their empathy could diminish the probability of cyberbullying perpetration (Zych et al., 2019). It is important to note that contrary to traditional bullying, in the case of cyberbullying the bully does not see the victim and their reactions, and consequently, empathy holds back the bully even less (Slonje & Smith, 2008). Lastly, cyberbullying interventions could encourage bystanders’ and non-experienced participants’ active-defending behaviour, as the outcome of cyberbullying incidents are strongly determined by the reaction of bystanders. Bystanders can stay passive or they can also take an active role, as they can defend the victim, or they can join the bully (Burton et al., 2013; Pozzoli & Gini, 2010).

According to the meta-analysis of Gaffney et al. (2019), cyberbullying intervention and prevention programmes reduce the involvement in cyberbullying by 10–15% and victimisation by 14%. The long-term effect of anti-cyberbullying programmes is questionable. Some of the reviews report a weak or an insignificant effect (Cantone et al., 2015; Lan et al., 2022), others do not find association between programme duration and effectiveness (Ng et al., 2022). Judging the effectiveness of cyberbullying intervention programmes is hard not only because of the variability of these programmes, but also due to the low number of reliable, scientifically founded programmes with follow-up measurements (Della Cioppa et al., 2015). Based on the findings so far, intervention programmes that involve parents and the entire school community are more effective, as the phenomenon of cyberbullying has an extended impact on more levels—individual, family, and school (Cantone et al., 2015; Hutson et al., 2018). Since victims of cyberbullying turn mostly to their friends, programmes concentrating not only on the victims, but on the bystanders, the network around the victims as well can be more effective (Cassidy et al., 2013).

Accordingly, some of the cyberbullying prevention programmes include peer-educators (e.g., CyberMentors—Von Kaenel-Flatt & Douglas, 2012; Noncadiamointrappola—Menesini et al., 2012; MARC—McCoy et al., 2017). Apart from the general benefits of peer-education (Turner & Shepherd, 1999), involving peers in cyberbullying intervention programmes can prove to be especially fruitful. Peers are more up to date in the digital world and they are familiar with the applications and platforms popular among younger generations and can thus be more authentic educators than adult/older teachers. Nevertheless, how effective anti-bullying peer education programmes are is unclear. In their meta-analysis on bullying intervention programmes, Farrington and Ttofi (2009) found that involving peer educators increases victimisation. However, according to Smith et al. (2012) the former conclusion was confounded, as peer education programmes can largely differ regarding the involvement of peer educators, the length and frequency of intervention, the methods used, etc., and thus their effectiveness can also be different. Namely, the relative increase in victimisation was produced not by the presence of peer educators, but the characteristics of programme delivery. Correspondingly, in their updated systematic review, Gaffney et al. (2021) do not make a distinction between school-based programmes involving or not involving peer-educators regarding the effectiveness of the programme.

### The STAnD Peer Education Programme

The STAnD peer education programme (the acronym of Study, Teach, Understand)—among various health issues—focuses on the subject of internet security and cyberbullying (Feith et al., 2018). The programme was developed by the team of Health Education with Peer Education supported by the Hungarian Academy of Sciences and aims to increase health-awareness among children and adolescents while interconnecting multiple levels of the educational system. University students from the fields of health sciences and pedagogy, trained and tutored by professionals, implement health education programmes for primary and secondary school students. The STAnD programme is based on theories related to peer education (Social Learning Theory—Bandura, 1977; Differential Association Theory—Sutherland & Cressy, 1960; Diffusion of Innovation Theory—Rogers, 1983). The anti-cyberbullying and internet security programme intended to inform children about safe internet use, especially highlighting the danger of internet addiction and the vulnerability to cyberbullying. The programme did not focus on diminishing the time of internet use, but rather to spotlight its risks and benefits. Regarding cyberbullying the programme aimed to define the phenomenon itself and to raise children’s awareness of the possible consequences. It provided information about organisations aiding in a bullying situation and thus explaining the importance of help-seeking. Talking through cyberbullying cases, alternative reactions were discussed. By sensitising children, the indirect goal – in accordance with the former research results (Dooley et al., 2010; Pozzoli & Gini, 2010; Zych et al., 2019) – was to decrease the prevalence of cyberbullying: to ensure that the victims ask for help, the bystanders intervene, and the bullies change their attitude.

In the course of the STAnD anti-cyberbullying programme, students of Semmelweis University, Faculty of Health Sciences and Eötvös Loránd University, Faculty of Primary and Pre-School Education along with volunteer secondary school students carried out interactive, 3-h-long (1 session, altogether 4 × 45 min) programmes about internet security and cyberbullying in 5 schools among 8–15-year-old students. Generally, 4 university students from the fields of health sciences (2) and pedagogy (2) and 1 secondary school student created a peer-educator group working with 20–25 participants (by school class) supervised by 1 tutor.

University and secondary school students applied to the STAnD programme through a motivational letter. Students prepared for their role as peer-educators on an elective university course (5 occasions, 24 contact hours). This course introduced them to the STAnD programme, the concept of peer-education, various pedagogical methods, and of course, they got familiar with the issue of cyberbullying including other anti-cyberbullying programmes. During this course peer-educators created their own programme-plan based on a detailed discussion on aims with direct and indirect activity goals (e.g., knowledge transfer, sensitisation, awareness raising) in each topic. This planning stage enabled peer-educators to be creative while keeping the health-education programme in a standard frame. Completion of this course was a pre-requisite to participating in the programme as a peer-educator.

In the course of the 3-h-long STAnD internet security and cyberbullying programme, after a short team-building, peer-educators discussed the main issues of digitalisation, internet security and internet addiction with the participants. The phenomenon of cyberbullying was defined together with the children, and the possible consequences were discussed as well. STAnD programme intended to decrease the prevalence of cyberbullying in 3 ways.

1. In order to encourages *willingness to ask for help*—especially in the case of victims—peer-educators presented several helplines, organisations, websites, etc. where participants can find specific help in the case of cyberbullying. Besides knowledge-transfer, peer-educators discussed with the participants from whom and why is it important to ask for help.
2. Peer-educators encouraged participants to intervene in a case of cyberbullying, and instead of a *passive reaction*, take an *active-defending reaction*, namely, defend the victim*.* For sensitising students, cyberbullying cases were analysed with the participants through dramatic play,^2^ considering alternative reactions.
3. *Empathy for victims* was increased through dramatic play,^3^ as well, spotlighting the emotions of victims in a cyberbullying situation.

After debating the most important netiquette rules, children created ‘I-codex’ in small groups. Intervention was closed by summarising the main messages. Peer-educators designed and applied interactive and playful activities, which differed by age-group. Beside the participants and peer-educators, only tutors were present during the anti-bullying intervention programme.

## Aims

The aim of the study is to present the effects of our anti-cyberbullying programme with peer-education on the short and the long run among lower and upper primary school students, regarding the participants’ cyberbullying roles. The effects of the programme were analysed in three dimensions:

1. Measurement of cyberbullying involvement through three cyberbullying types and identifying the cyberbullying roles (experienced/non-experienced, within experienced group: victim, bully, bully/victim, bystander).
2. Investigation of changes in *willingness to ask for help, empathy for victims*, and *active-defending or passive reaction* among the participants by school class (lower/upper primary) and involvement in cyberbullying.
3. Satisfaction with the programme by school class (lower/upper primary) and involvement in cyberbullying.

As cyberbullying interventions have concentrated less on lower primary school-aged children so far (Cantone et al., 2015; Ey et al., 2015), the effectiveness of the programme was measured among lower and upper primary school participants. Based on former research results we hypothesised that effects of the programme and satisfaction with the programme will differ by school class (Salmivalli & Poskiparta, 2012; Smith, 2009). We presumed that age could be an important aspect when designing an anti-cyberbullying intervention. Involvement in cyberbullying was also analysed, since it can be hypothesised that the programme has different effects on the students depending on their involvement and roles in cyberbullying (Völlink et al., 2013). We intended to explore whether our anti-cyberbullying programmes affect the experienced and non-experienced groups differently.

## Method

Self-administered paper and pen survey was applied among the participants. Besides basic socio-demographical questions, questionnaire items measured involvement in cyberbullying and cyberbullying behaviour, as well as the level of satisfaction with the STAnD programme. Because of the special features and the special target group of STAnD programme, the items of the questionnaire were developed by a professional board (including psychologists, pedagogical researchers, teachers, and sociologists) based on a literature review.

Involvement in cyberbullying was measured by looking at the occurrence of the three types of cyberbullying in the 6 months before the data-collection^4^ [(1) Spreading secrets, rumors, personal information without permission: *denigration*; (2) Sending harassing, false messages: *flaming*; (3) Taking photos without permission to put somebody at a disadvantage: *outing*]. Respondents could choose whether they experienced cyberbullying or not and in which role (victim, bully, bully/victim, bystander). We choose to measure cyberbullying involvement through multiple and specific cyberbullying forms and analysed them separately to get more valid results (Vandebosch & Van Cleemput, 2009). The measured cyberbullying types were selected based on their prevalence in the examined age-group in Hungary (Parti et al., 2018; Várnai et al., 2020).

Participants’ cyberbullying behaviour was measured in three dimensions.

1. Participants could mark whether or not they would ask for help in the case of cyberbullying on a 5-point scale^5^ (1 = would never ask for help, 5 = would always ask for help).
2. Participants could evaluate to what extent victims are affected by the three types of cyberbullying on a 10-point scale^6^ (1 = not affected at all, 10 = gravely affected).
3. Participants’ engagement in cyberbullying was examined in three fictitious situations: Situation A: happy slapping,^7^ Situation B: exclusion,^8^ Situation C: denigration^9^). Responses (including the texts under the option “Other”) were categorised into active-defending and passive, the uncategorizable answers being excluded from the analysis.

Regarding the evaluation of the programme, we investigated (1) how students evaluate the programme^10^; (2) are they willing to participate in further programmes,^11^ (3) do they recommend it to their friends.^12^ The study used a non-randomised uncontrolled study design.

As we intended to capture the long-term effects of our programme, participants were surveyed three times: (1) one week before the STAnD programme (2019 April) (Baseline), (2) right after the STAnD programme (2019 April) (T1), and (3) 6 months after the STAnD programme (2019 October) (T2). The units of the analysis were participants as individuals. Altogether 933 students participated in the programme, and filled out the survey before (Baseline) and right after the intervention (T1). Due to the COVID-19 pandemic we could not finish the 3^rd^ wave of the data collection in all the 5 schools. At the follow-up measurement (T2) 536 participants (from 3 schools) completed the survey. Only those students were included in the analysis, who participated in all the three data-collections (535, 57.3%). In order to interconnect students’ three questionnaires from the three data-collections, we asked the participants to give their kindergarten sign, and their birth month and day. Based on this information we were able to identify students’ questionnaire and interconnect them for the analysis without offending the anonymisation. Participants filled the pen and paper survey independently in classrooms. In the case of the first and third data-collection in the attendance of a teacher in a designated time, while right after the programme in the attendance of peer educators and tutors.

Schools were recruited by self-selection. The selection criteria for the 5 schools were that school board members should be committed to the research, and parents should contribute to their children’s participation. The parents were informed about the project by a letter, they gave written consent on a voluntary basis, and they had the right to refuse the participation of their children at any time. Participants also had the possibility to refuse to participate, any time during the programme. Our research is ethically acceptable following the World Medical Association’s Declaration of Helsinki and the requirements of all applicable local and international standards. The Semmelweis University Regional and Institutional Committee of Science and Research Ethics has approved this research (SE-TUKEB No. 230/2017).

### Statistical Methods

Descriptive statistics (frequencies and proportions) were used to describe the study sample. To compare the means of lower and upper primary school students on the dependent variables measured, we used independent samples t-tests with Cohen’s d effect size. To examine associations between school classes and involvement in cyberbullying, Pearson’s chi-square test was calculated with phi/Cramer’s V effect size. In order to evaluate the effect of the peer education programme, we used repeated measures ANOVA with within-subjects variables (Time: baseline, after the intervention—T1, six months later—T2) and between-subjects factor (Cyberbullying involvement—experienced versus non-experienced; Cyberbullying roles—victim, bully, bully/victim, bystander). We analysed the Time main effect and Time × Involvement/Roles interactions (Simple effects) with the calculation of partial eta squared effect size. The effects of the programme on the outcome variables are summarised in Supplementary Table 2–Table 4. For all tests the significance level was set to 5%. IBM SPSS Statistics for Windows, version 25.0 (IBM Corp., Armonk, N.Y., USA) was used for statistical analysis.

## Results

### Characteristics of the Sample

The mean age of the students was 11.22 years (SD = 1.57, min = 8, max = 15). 36.4% of them were (n = 195) from lower primary school, 63.6% (n = 341) from upper primary school, and more than half of them were girls (54.7%, n = 293). Most of the students (78.7%, n = 417) rated their school performance as excellent/good, 19.1% (n = 101) of them had average and only 2.2% (n = 12) had fair/poor performance. It is important to note that great majority of the students i.e. 93.9% (n = 412) had parents whose highest level of education was a bachelor’s or higher degree.

### Involvement in Cyberbullying

Regardless of students’ school class, the most frequently experienced cyberbullying was spreading secrets, rumors, or personal information without permission (Type 1 denigration). A further one-third of upper primary school students experienced the sending of harassing, false messages (Type 2 flaming), as well as photos taken without permission to put somebody at a disadvantage (Type 3 outing). These latter two types of cyberbullying were found to be far less common among lower primary school students (Table 1).

**Table 1:** Cyberbullying involvement among lower and upper primary school students

Cyberbullying types	Cyberbullying involvement	Lower primary school (n = 189)		Upper primary school (n = 327)		χ^2^	*p*	Ф/V
		n	%	n	%			
1. Denigration	Non-experienced	132	69.8	209	63.9	1.877	0.178	0.06
	Experienced	57	30.2	118	36.1			
	Victim	21	36.8	26	22.0	9.870	0.020	0.24
	Bully	11	19.3	13	11.0			
	Bully/victim	6	10.5	30	25.4			
	Bystander	19	33.3	49	41.5			
2. Flaming	Non-experienced	154	81.5	218	66.7	13.066	< 0.001	0.16
	Experienced	35	18.5	109	33.3			
	Victim	6	17.1	30	27.5	na	na	na
	Bully	2	5.7	9	8.3			
	Bully/victim	2	5.7	9	8.3			
	Bystander	25	71.4	61	56.0			
3. Outing	Non-experienced	165	87.3	232	70.9	18.053	< 0.001	0.19
	Experienced	24	12.7	95	29.1			
	Victim	8	33.3	20	21.1	na	na	na
	Bully	0	0.0	9	9.5			
	Bully/victim	1	4.2	13	13.7			
	Bystander	15	62.5	53	55.8			

Cyberbullying roles revealed a significant association with school class in Type 1. The bully/victim role was more common among upper primary school students than in lower primary school. In Types 2 and 3, we did not evaluate the association statistically because the expected cell count assumption was violated. Based on the frequencies, it is important to note that although in either school class most students did not engage in these types of cyberbullying, the majority of the children affected participated in certain incidents as a bystander (Table 1).

### The Effects of the Programme by Involvement in Cyberbullying

During data analysis, we looked at lower and upper primary school students separately when analysing their involvement in the three types of cyberbullying. First, we compared experienced and non-experienced groups. Within the experienced group, the cyberbullying roles (victim, bully, bully/victim, bystander) were analysed many times using only descriptive statistics due to a low case number.

#### Willingness to Ask for Help

**Fig. 1:**
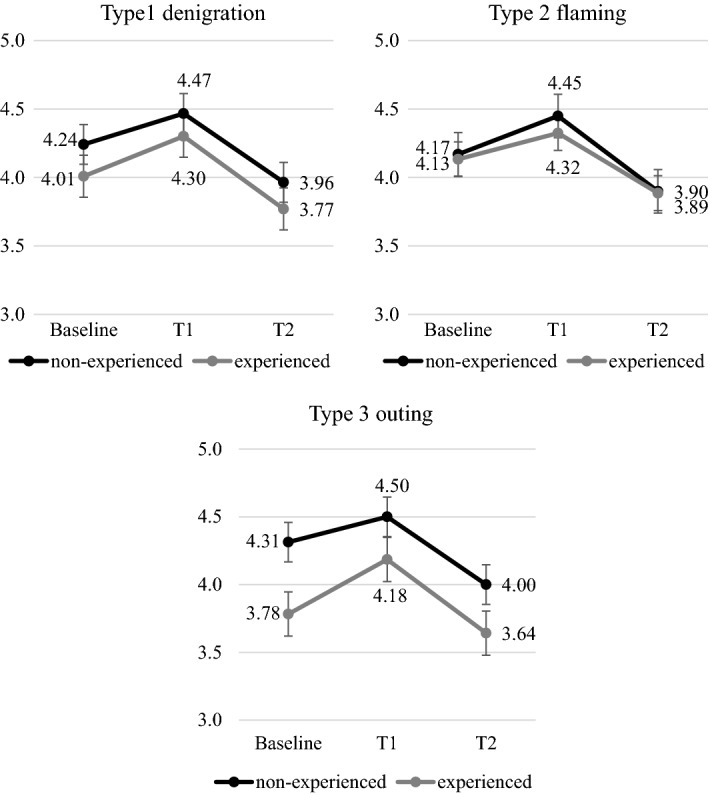
Changes in *willingness to ask for help* in upper primary students by involvement in cyberbullying [x-axis: Time, y-axis: mean, error bars: standard error, Type 1: F(2,620) = 32.412, *p* < 0.001, $\eta_{\rm p}^{2}$ = 0.10, Type 2: F(2,620) = 28.727, *p* < 0.001, $\eta_{\rm p}^{2}$ = 0.09, Type 3: F(2,620) = 30.027, *p* < 0.001, $\eta_{\rm p}^{2}$ = 0.09]

At Baseline, most students said they “would always ask for help” (52.9%, n = 270) or “would rather ask for help” (22%, n = 112), regardless of school class. There were significant changes in Time main effect on *willingness to ask for help* both in lower and upper primary school students. Upper primary students showed a significant increase in *willingness to ask for help* after the intervention (T1), while compared to this, six months later (T2) their *willingness to ask for help* decreased. These changes were irrespective of experiences in all three types of cyberbullying (Fig. 1).

**Fig. 2:**
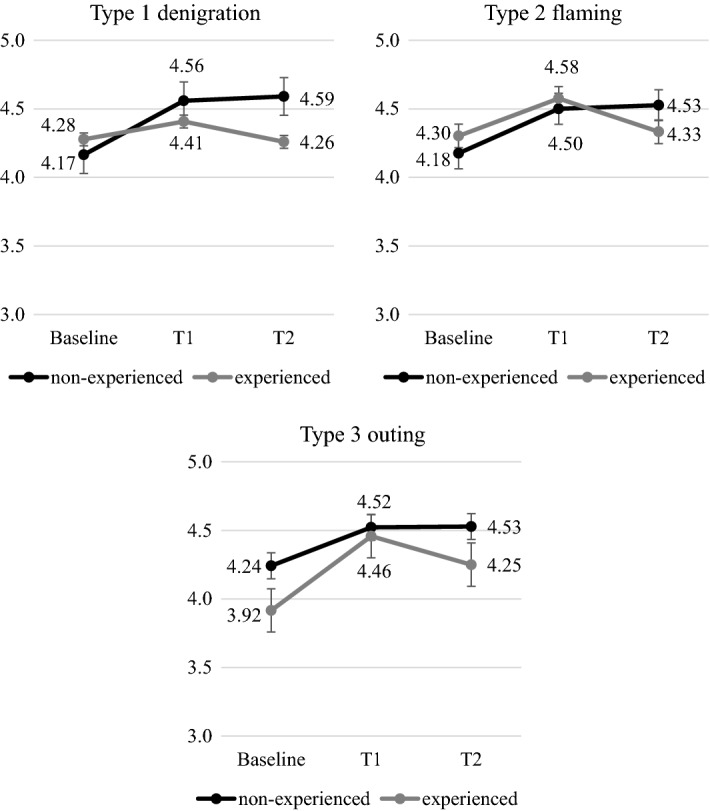
Changes in *willingness to ask for help* in lower primary students by the involvement in cyberbullying [x-axis: Time, y-axis: mean, error bars: standard error, Type 1: F(2,358) = 4.577, *p* = 0.011, $\eta_{\rm p}^{2}$ = 0.03, Type 2: F(2,358) = 3.7910, *p* = 0.021, $\eta_{\rm p}^{2}$ = 0.02, Type 3: F(2,358) = 6.032, *p* = 0.003, $\eta_{\rm p}^{2}$ = 0.03]

Examining changes in lower primary students, Time × Cyberbullying involvement interaction was significant in Type 1 (denigration) [F(2,358) = 3.021, *p* = 0.032, $\eta_{\rm p}^{2}$ = 0.03]. Based on the simple effects, the experienced group revealed a non-significant change in Time. However, the *willingness to ask for help* statistically increased in the non-experienced group, and this change remained after six months later. In Types 2 (flaming) and 3 (outing), *willingness to ask for help* showed a statistically non-significant Time × Cyberbullying involvement interaction; at the same time, we can see a trend similar to Type 1 (denigration) in the non-experienced group. In these types of cyberbullying, the experienced group showed an increase after the intervention and a decrease six months later in the *willingness to ask for help*, similarly to upper primary students (Fig. 2). Within the experienced group, the cyberbullying roles (victim, bully, bully/victim, bystander) had no significant effects on the changes of the *willingness to ask for help.*

#### Empathy for Victims

At Baseline, students rated rather high the extent victims are affected by cyberbullying (7 or higher) and there was a significant difference between lower and upper primary school students in Type 3 (outing) [t(513) = 4.135, *p* < 0.001, d = 0.40] and in Type 2 (flaming) [t(513) = 3.254, *p* = 0.001, d = 0.30]. Lower primary students rated the extent victims are affected by cyberbullying higher than upper primary students did. In Type 1 (denigration), there was a non-significant difference between the school classes [t(513) = 1.282, *p* = 0.200].

**Fig. 3:**
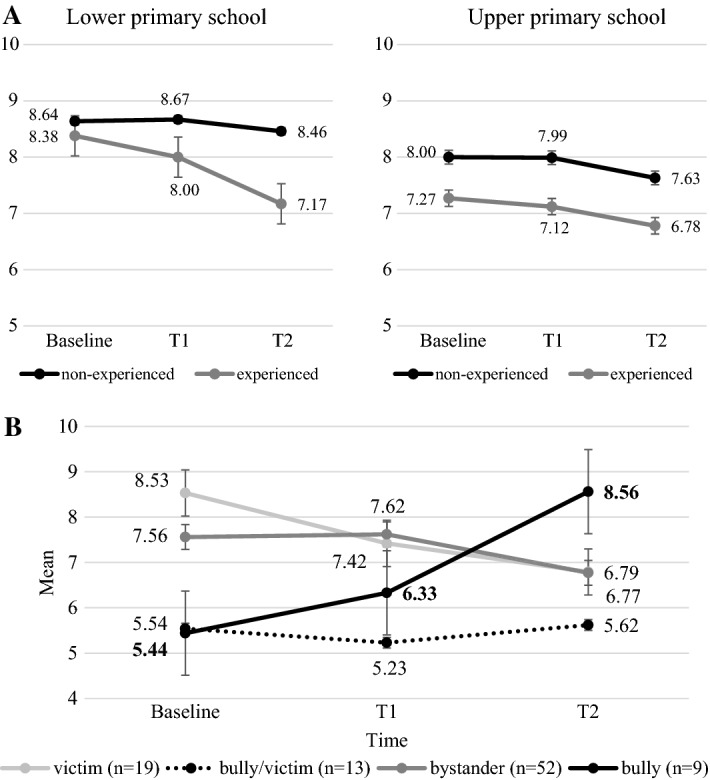
**A** Changes in estimating the extent victims are affected by cyberbullying in primary school students by involvement in cyberbullying Type 3 (outing) (x-axis: Time, y-axis: mean, error bars: standard error). **B** Changes in estimating the extent victims are affected by cyberbullying in upper primary students by cyberbullying roles (x-axis: Time, y-axis: mean, error bars: standard error)

In Type 3 (outing), both lower [F(2,368) = 3.292, *p* = 0.038, $\eta_{\rm p}^{2}$ = 0.02] and upper [F(2,634) = 3.624, *p* = 0.027, $\eta_{\rm p}^{2}$ = 0.01] primary school students showed a slight significant decrease six months later in estimating the extent victims are affected by cyberbullying. It is important to note that this decrease was more pronounced in lower primary students who experienced cyberbullying. However, Time and Cyberbullying involvement showed a non-significant interaction [lower primary: F(2,368) = 1.655, *p* = 0.191, $\eta_{\rm p}^{2}$ = 0.01; upper primary: F(2,634) = 0.101, *p* = 0.904, $\eta_{\rm p}^{2}$ = 0.00] (Fig. 3).

**Fig. 4:**
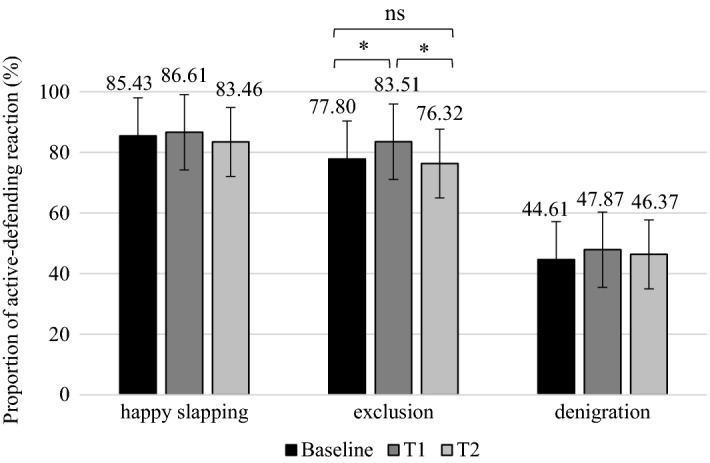
Changes on cyberbullying engagement (error bars: standard error) *p < 0.05, ns: non-significant

Examining the cyberbullying roles within the experienced group, we revealed significant interaction effects in upper primary students [F(6,178) = 3.212, *p* = 0.005, $\eta_{\rm p}^{2}$ = 0.10]. Based on the simple effects, the declining trend described above (Fig. 3) was observed in the victim and bystander roles. The bully/victim role, which showed the smallest score, had a non-significant change. The bully role showed a significant increase in estimating the extent victims are affected by cyberbullying after the intervention and six months later (Fig. 4).

In Type 1 (denigration) and in Type 2 (flaming) neither lower nor upper primary students showed a significant change in estimating the extent victims are affected by cyberbullying during the measurements, and this was not influenced by Involvement in cyberbullying either (Descriptive statistics see Supplementary file 1).

#### Engagement in Cyberbullying

At Baseline lower and upper primary students showed a significant difference in each of the three fictitious situations. In the situation of happy slapping and exclusion a larger number of lower primary students would have an *active-defending reaction*, than upper primary students. In the situation of denigration, students would have an *active-defending reaction* the least, although, compared to lower primary students, a larger proportion of upper primary students would be active (Table 2).

**Table 2:** Active-defending reaction at Baseline in lower and upper primary school students

	Lower primary school		Upper primary school		t	*p*	d
	M*	SD	M	SD			
Happy slapping	91.07	28.64	81.38	39.06	2.294	0.023	0.28
Exclusion	85.39	35.42	73.33	44.30	3.272	0.001	0.30
Denigration	34.59	47.72	50.00	50.09	-3.171	0.002	0.32

*%

*Active-defending reaction* measured during the programme (at T1 and T2) showed a significant change in Time [F(2,942) = 5.853, *p* = 0.003, $\eta_{\rm p}^{2}$ = 0.01] only at the situation of exclusion (Situation B) [F(2,942) = 5.853, *p* = 0.003, $\eta_{\rm p}^{2}$ = 0.01], regardless of school classes [F(2,942) = 0.232, *p* = 0.793]. Compared to Baseline (M = 77.80%, SD = 41.60), a significant increase could be detected at T1 (*p* = 0.007) in students who would have an *active-defending reaction* (M = 83.51, SD = 37.15). At T2 measurement, however, the number of students willing to have an *active-defending reaction* significantly decreased (*p* = 0.007) (M = 76.32, SD = 42.55), which did not differ from Baseline values (*p* = 1.000; Fig. 4).

In situations of happy slapping [F(2,504) = 0.696, *p* = 0.499] and denigration [F(2,794) = 0.480, *p* = 0.619] no statistically significant change in Time could be observed in the proportion of *active-defending reaction*. As for the number of students in the programme having an *active-defending reaction* in each situation, see Fig. 4.

### Programme Evaluation

Beside examining the changes regarding cyberbullying, we investigated how students evaluate the programme and if they were willing to participate in further programmes or to recommend it to their friends. Students’ satisfaction with the programme was analysed by the involvement in cyberbullying too.

Participants evaluated the programme and the peer-educators on a 16-item scale (Cronbach α = 0.897). On a scale of 18 to 80 the students participating evaluated the programme at an average of 67.03 points (SD = 10.37). Lower primary students (M = 71.89, SD = 8.99) were significantly more satisfied with the programme than upper primary students M = 64.44, SD = 10.14) [t(457) = 8.085, *p* < 0.001, d = 0.78].

In sum, more than the two-thirds of the students (65.2%, n = 349) indicated that they would take part in similar programmes and only 4.9% gave a negative answer (n = 26). Lower primary students were more willing to participate in such programmes (76.4%, n = 149) [χ^2^(2,N = 535) = 18.040, *p* < 0.001, Cramer V = 0.18], compared to upper primary students (58.8%, n = 200), the one-third of whom selected the answer “I don’t know” (34.7%, n = 118).

Finally, also more than the two-thirds of students (67.2%, n = 358) would recommend the programme to their friends. School class in this respect also showed a significant association [χ^2^(2,N = 533) = 18.138, *p* < 0.001, Cramer V = 0.18]. A larger number of lower primary students (78.5%, n = 153) would recommend the programme, compared to upper primary students (60.7%, n = 205), with almost the one-third of whom giving an uncertain answer (32%, n = 108).

Cyberbullying involvement and roles (experienced, non-experienced, and victim, bully, victim/bully, bystander) did not significantly affect the satisfaction with the programme. Participants reported a relatively high satisfaction regardless of their involvement and roles in cyberbullying.

## Discussion

In our study we analysed the short- and long-term effects of a pilot cyberbullying programme within the framework of the STAnD health education project among lower and upper primary school students, with a special focus on children’s involvement in cyberbullying. We are aware that a one-time and short, 3-h intervention is not sufficient for having a long-term effect on participants’ cyberbullying behaviour. Nevertheless, it is worth investigating what changes can be observed after a one-time and short, 3-h intervention and which groups of participants can be addressed with more success on such an intervention. The effects of the programme were evaluated in terms of the students’ *willingness to ask for help*, *empathy for victims*, and *cyberbullying engagement* with regards to the dimensions of school classes and involvement in cyberbullying.

STAnD programme aimed at shaping the cyberbullying behaviour of the students participating in three aspects. Research so far (Dooley et al., 2010; Smith et al., 2008; Spears et al., 2015) points at victims’ decreased *willingness to ask for help*, therefore, one of the main goals of the intervention was to change this. *Willingness to ask for help* increased in both age-groups, although six months after the intervention only lower primary students’ increased level remained unchanged in the non-experienced group, while upper primary students’ willingness fell back to the level at baseline. This result is in line with former research showing that victims and bully-victims employ different coping strategies, compared to non-experienced children (Smith et al., 2008; Völlink et al., 2013). Unlike victims and bully-victims, non-experienced children are willing to ask for help. This is why it is important to involve non-experienced children to anti-cyberbullying programmes as potential bystanders, who may stop the cyberbullying situation.

Another goal of the programme was to increase students’ *empathy for victims* in order to decrease cyberbullying prevalence. During the three measurements, generally no apparent change could be detected in students’ estimation of the extent victims are affected by cyberbullying. At the same time, it is important to note that bullies from upper primary school showed an increase in estimating the extent victims are affected by cyberbullying on victims after the intervention, which could also be observed six months later. This demonstrates that even bullies can be addressed in such intervention programmes. However, it is important to note that according to our baseline measurements, most of the students are fundamentally aware of how gravely cyberbullying affects the victims.

The third goal in changing students’ cyberbullying behaviour was to reinforce their *active-defending reaction* in cyberbullying situations. After the STAnD programme, in situations of exclusion, *active-defending reaction* increased among the participants, although at the measurement six months after the programme they showed the same behaviour as at the baseline.

In conclusion, the effects of our 3-h peer education intervention could be detected in the increased *willingness to ask for help* and reinforced yet not long-lasting *active-defending reaction*. Meta-analyses of anti-cyberbullying programmes reported very small to small, modest effects (Gaffney et al., 2019; Lan et al., 2022; Ng et al., 2022; Polanin et al., 2022). The effectiveness of the STAnD programme was small to moderate in the short run, and very small, small in the long run. These effects are smaller than previous studies reported effectiveness regarding empathy and active-defending behaviour, although the direct comparison is limited due to the different methodology, intervention, and measured outcomes (Garandeau et al., 2022; Vlaanderen et al., 2020; Wang, 2021).

Our pilot programme imply that one-time and short anti-cyberbullying programmes are not effective (Cantone et al., 2015; Lan et al., 2022). Our results indicate that in order to reach long-term effects, longer programmes and recurring sessions (at least after 6 months) are necessary with a possible involvement of teachers and the students’ social environment (peers, family) (Gaffney et al., 2019).

As our findings show, attitude to cyberbullying can be better improved among lower primary school students and with longer lasting effects than in upper primary students. Six months after the programme, lower primary students had preserved their *willingness to ask for help* more, although regarding their *empathy for victims*, no detectable change could be observed. At the same time, the extent victims are affected by cyberbullying was rated higher in all the three types of cyberbullying examined. The students’ *active-defending reaction* was not long-lasting in either group, yet in situations of happy slapping and exclusion lower primary students were found to be more willing to protect the victims, compared to upper primary students.

Even though the number of cyberbullying intervention programmes targeting primary school students is scarce (Cantone et al., 2015; Ey et al., 2015), our findings pinpoint that even a one-time intervention (STAnD programme) can reach better results in lower primary than in upper primary school students, although over a six months’ period of time this effect weakened in both groups. In terms of openness to the programme, lower primary students were more satisfied, would participate in similar programmes and would recommend it to their friends more likely than students in upper primary school. The success of antibullying programmes among younger children are explained mainly by developmental changes, namely, they accept authority better than older teenagers who are more critical and less rule-abiding in general (Smith, 2009). Therefore, results spotlight that anti-cyberbullying programmes should be age-appropriate. Although students’ involvement in cyberbullying was less prevalent in lower primary compared to upper primary school and different cyberbullying types are typical in the two groups, we can see that the phenomenon of cyberbullying affects this age group, as the majority of them has access to the internet via their smart devices in their everyday life (Ey et al., 2015). Therefore, it is of utmost importance to reach out to this age group with cyberbullying intervention programmes, which can thus probably decrease the prevalence of cyberbullying in later life.

The effects of our pilot programme were assessed with regards to involvement in cyberbullying, although due to the low case numbers in the groups of victim, bully, victim/bully and bystander only a few conclusions could be drawn. Our findings show that such programmes may affect the experienced and non-experienced groups in different ways. Students’ cyberbullying role did not influence their satisfaction with the programme, so we assume that we could address the participants regardless of their level of involvement in cyberbullying. Previous studies also agree that those interventions can be truly effective which focus not only on the victim and the bully but on their wider environments (Cantone et al., 2015; Hutson et al., 2018). It is important to reach the whole school community and possibly the families as well (Farrington & Ttofi, 2009; Cassidy et al., 2013). Accordingly, the STAnD programme targets whole school classes and, where feasible, whole school communities. The findings of our pilot programme underline that we could address students both with and without cyberbullying experience. Peer relationships and their social support play a vital role in the outcome of cyberbullying incidents and in the intensity of their impacts (Burton et al., 2013; Pozzoli & Gini, 2010).

Although we cannot compare the effectiveness of different cyberbullying intervention programmes in this present study, knowing how vital the role of bystander is in the outcome of cyberbullying situations, we believe that involving peer educators—despite critical views in this matter (Farrington & Ttofi, 2009)—can be effective. With proper supervision and preparation, peer educators can have substantial effect on their peers, especially if they come from the students’ class or school (Menesini et al., 2012; Smith et al., 2012; Von Kaenel-Flatt & Douglas, 2012; McCoy et al., 2017). Such peer education programmes in health education are not only cost-effective but can also have long-term effects (Turner & Shepherd, 1999).

One of the limitations of our study is the lack of a control group. Although the observed outcomes would be more valid in a research design with a control group, based on ethical and pedagogical considerations, our research group chose to include every participant in the intervention. As our study was a non-randomised, uncontrolled study design, thus interpretation of the effectiveness of the programme is limited.

## Conclusion

In our study we examined the effects of a pilot programme on cyberbullying. The students in the programme showed positive changes in their *willingness to ask for help* and their *active-defending reaction* after the programme, but this effect was smaller or there was no effect six months later. Nevertheless, our findings underline that long-term effects in health promotion cannot be achieved with one-time and short interventions (Gaffney et al., 2019). A more substantial and lasting effect could be observed among lower primary school students and their openness to the programme was also higher than in upper primary students. Anti-cyberbullying programmes should take into consideration participants’ involvement and roles in cyberbullying.

## Supplementary Information

Below is the link to the electronic supplementary material.

Supplementary file1 (DOCX 24 kb)

## Footnotes

1 The most substantive elements of the definition of cyberbullying are that it is an aggressive, intentional, repetitive act performed by a group or an individual using electronic or digital media (Smith et al., 2008; Tokunaga, 2010).

2 e.g., One of your classmates excluded another classmate from the Viber/Messenger etc. group of your school class. What would you do? What could you do to defend your classmate?

3 e.g., Someone uploads a disadvantageous picture about one of your classmates to the social media with a rude comment. How does he/she feel him/herself? What could he/she do? What could be the intention of the bully? How does the bully feel him/herself? What would you do as a bystander?

4 Have these situations happened to you in the past 6 months? You can select multiple answers. Others did it to me/I did it to others/Others did it to me and I did it to others/I wasn’t affected personally, but I was witnessing others doing it/I didn’t experience it. (Domonkos, 2014; Zsila, 2018)

5 Would you ask for help in the case of cyberbullying? Yes, I would, in every case/Yes, I would/I don’t know/No, I wouldn’t/No, I surely wouldn’t.

6 In your opinion, to what extent do these acts cause pain for somebody who is a victim? 1) Spreading secrets, rumours, personal information without permission; 2) Sending harassing, false messages; 3) Taking photos without permission to put somebody at a disadvantage. (Domonkos, 2014; Zsila, 2018)

7 At the school yard two children are fighting with each other, while a third one is video recording with a phone intending to spread the video to others. Would you ask the child taking a video record to stop it? Yes, sure/Maybe/I wouldn’t do anything because he/she could do the same with me/No, it’s not my job/Other:…….

8 One of your classmates exclude another one from the Viber/Facebook etc. group of your school class based on a personal matter. What would you do in this situation as a bystander? I put him/her back to the group immediately/I would ask to put him/her back to the group/I wouldn’t do anything, because they may exclude me, as well/I wouldn’t do anything, because it’s not my job/Other:…….

9 One of your classmates likes another one. He/she shares this secret with his/her best friend. His/her best friend writes this secret to the Viber/Facebook etc. group of your school class. What would you do in the shoes of this boy/girl, whose secret was revealed? Please, read all the answers and choose what you would do mostly. I would share his/her secret, too, out of revenge/ I wouldn’t spend time with him/her/ I would immediately (online or by phone) tell him/her that he/she humiliated me/I would tell him/her face to face that he/she humiliated me/I would never tell my secret anymore/I would leave the group/It wouldn’t bother me/I wouldn’t do anything because I would be afraid that they ban me/Other:……

10 Participants evaluated the programme and the peer-educators on a 16-item scale (Cronbach α = 0.897).

11 Are you willing to participate in similar programmes? Yes/No/I don’t know.

12 Would you recommend this programme to your friends? Yes/No/I don’t know.
