# Supplementary material for: The Effects of a Cyberbullying Intervention Programme Among Primary School Students
Source: Child Youth Care Forum. 2022 Oct 2;52(4):893–911. doi: 10.1007/s10566-022-09714-9 (PMC9527071; doi:10.1007/s10566-022-09714-9)
Supplement: Supplementary file 1 — Supplementary file1 (DOCX 24 kb) [file 10566_2022_9714_MOESM1_ESM.docx]

**Supplementary files**

Table 1. Changes on empathy for victims in lower and upper primary students by involvement in cyberbullying in Type 1 (denigration) and Type 2 (flaming)

| Type 1 denigration |  | Lower primary school | | | Time  (Baseline-T1-T2) | | | Time x Involvement of cyberbullying | | |
| --- | --- | --- | --- | --- | --- | --- | --- | --- | --- | --- |
|  |  | Baseline M(SD) | T1  M(SD) | T2  M(SD) | F | p | η^2^_p_ | F | p | η^2^_p_ |
|  | non-experienced | 7.78(2.56) | 8.32(1.85) | 7.95(2.22) | 0.855 | 0.426 | 0.01 | 0.682 | 0.506 | 0.00 |
|  | experienced | 7.36(2.15) | 7.38(1.76) | 7.30(2.49) |  |  |  |  |  |  |
|  |  | Upper primary school | | | Time  (Baseline-T1-T2) | | | Time x Involvement of cyberbullying | | |
|  |  | Baseline M(SD) | T1  M(SD) | T2  M(SD) | F | p | η^2^_p_ | F | p | η^2^_p_ |
|  | non-experienced | 7.41(2.30) | 7.61(2.21) | 7.33(2.12) | 1.246 | 0.288 | 0.00 | 1.595 | 0.204 | 0.01 |
|  | experienced | 7.42(2.39) | 7.09(2.23) | 7.03(2.07) |  |  |  |  |  |  |
| Type 2 flaming |  | Lower primary school | | | Time  (Baseline-T1-T2) | | | Time x Involvement of cyberbullying | | |
|  |  | Baseline M(SD) | T1  M(SD) | T2  M(SD) | F | p | η^2^_p_ | F | p | η^2^_p_ |
|  | non-experienced | 8.26(2.19) | 8.17(1.90) | 8.04(2.28) | 2.291 | 0.061 | 0.02 | 1.596 | 0.204 | 0.01 |
|  | experienced | 7.74(2.77) | 8.09(2.08) | 7.03(3.02) |  |  |  |  |  |  |
|  |  | Upper primary school | | | Time  (Baseline-T1-T2) | | | Time x Involvement of cyberbullying | | |
|  |  | Baseline M(SD) | T1  M(SD) | T2  M(SD) | F | p | η^2^_p_ | F | p | η^2^_p_ |
|  | non-experienced | 7.53(2.55) | 7.49(2.27) | 7.30(2.23) | 1.069 | 0.344 | 0.00 | 0.798 | 0.450 | 0.00 |
|  | experienced | 7.33(2.38) | 6.96(2.38) | 7.15(2.07) |  |  |  |  |  |  |

Table 2. The effect of the programme by involvement in cyberbullying on *willingness to ask for help* among lower and upper primary students, * Mean (SD)

| Lower primary students | denigration | | | | | | | | | |
| --- | --- | --- | --- | --- | --- | --- | --- | --- | --- | --- |
|  | Baseline* | T1 | T2 | | | F | | p | | η^2^_p_ |
| Main effects (Time) | 4.20 (1.11) | 4.51 (0.86) | 4.49 (0.79) | | | 4.577 | | 0.011 | | 0.03 |
| Simple main effects | Baseline | T1 | T2 | | | F | | p | | η^2^_p_ |
| non-experienced | 4.17 (1.19) | 4.56 (0.85) | 4.59 (0.74) | | | 10.752 | | < 0.001 | | 0.08 |
| experienced | 4.28 (0.88) | 4.41 (0.88) | 4.26 (0.87) | | | 0.659 | | 0.520 | | 0.01 |
|  | flaming | | | | | | | | | |
|  | Baseline | T1 | T2 | | | F | | p | | η^2^_p_ |
| Main effects (Time) | 4.20 (1.11) | 4.51 (0.86) | 4.49 (0.79) | | | 3.910 | | 0.021 | | 0.02 |
| Simple main effects | Baseline | T1 | T2 | | | F | | p | | η^2^_p_ |
| non-experienced | 4.18 (1.14) | 4.50 (0.87) | 4.53 (0.76) | | | 8.974 | | < 0.001 | | 0.06 |
| experienced | 4.30 (0.95) | 4.58 (0.83) | 4.33 (0.92) | | | 1.165 | | 0.319 | | 0.04 |
|  | outing | | | | | | | | | |
|  | Baseline | T1 | T2 | | | F | | p | | η^2^_p_ |
| Main effects (Time) | 4.20 (1.11) | 4.51 (0.86) | 4.49 (0.79) | | | 6.032 | | 0.003 | | 0.03 |
| Simple main effects | Baseline | T1 | T2 | | | F | | p | | η^2^_p_ |
| non-experienced | 4.24 (1.13) | 4.52 (0.89) | 4.53 (0.76) | | | 6.641 | | 0.001 | | 0.04 |
| experienced | 3.92 (0.88 | 4.46 (0.66) | 4.25 (0.94) | | | 2.834 | | 0.069 | | 0.11 |
| Upper primary students | denigration | | | | | | | | | |
|  | Baseline | T1 | | T2 | F | | p | | η^2^_p_ | |
| Main effects (Time) | 4.16 (1.05) | 4.41 (0.95) | | 3.89 (1.21) | 32.412 | | < 0.001 | | 0.1 | |
| Simple main effects | Baseline | T1 | | T2 | F | | p | | η^2^_p_ | |
| non-experienced | 4.17 (1.19) | 4.56 (0.85) | | 4.59 (0.74) | 22.009 | | < 0.001 | | 0.1 | |
| experienced | 4.28 (0.88) | 4.41 (0.88) | | 4.26 (0.87) | 12.664 | | < 0.001 | | 0.1 | |
|  | flaming | | | | | | | | | |
|  | Baseline | T1 | | T2 | F | | p | | η^2^_p_ | |
| Main effects (Time) | 4.16 (1.05) | 4.41 (0.95) | | 3.89 (1.21) | 28.727 | | < 0.001 | | 0.09 | |
| Simple main effects | Baseline | T1 | | T2 | F | | p | | η^2^_p_ | |
| non-experienced (1) | 4.17 (1.04) | 4.45 (0.88) | | 3.90 (1.20) | 26.296 | | < 0.001 | | 0.11 | |
| experienced (0) | 4.13 (1.07) | 4.32 (1.07) | | 3.89 (1.24) | 8.648 | | < 0.001 | | 0.08 | |
|  | outing | | | | | | | | | |
|  | Baseline | T1 | | T2 | F | | p | | η^2^_p_ | |
| Main effects (Time) | 4.16 (1.05) | 4.41 (0.95) | | 3.89 (1.21) | 30.027 | | < 0.001 | | 0.09 | |
| Simple main effects | Baseline | T1 | | T2 | F | | p | | η^2^_p_ | |
| non-experienced (1) | 4.31 (0.93) | 4.50 (0.82) | | 4.00 (1.15) | 25.364 | | < 0.001 | | 0.10 | |
| experienced (0) | 3.78 (1.22) | 4.18 (1.17) | | 3.64 (1.31) | 10.734 | | < 0.001 | | 0.11 | |

Table 3. The effect of the programme by involvement in cyberbullying on *empathy for victims* among lower and upper primary students, * Mean (SD)

| Lower primary students | denigration | | | | | | |
| --- | --- | --- | --- | --- | --- | --- | --- |
|  | Baseline* | T1 | T2 | F | p | η^2^_p_ | |
| Main effects (Time) | 7.65 (2.45) | 7.76 (2.32) | 8.03 (1.87) | 0.855 | 0.426 | 0.01 | |
| Simple main effects | Baseline | T1 | T2 | F | p | η^2^_p_ | |
| non-experienced | 7.78 (2.56) | 8.32 (1.85) | 7.95 (2.22) | 2.391 | 0.094 | 0.02 | |
| experienced | 7.36 (2.15) | 7.37 (1.76) | 7.30 (2.49) | 0.022 | 0.978 | 0.00 | |
|  | flaming | | | | | | |
|  | Baseline | T1 | T2 | F | p | η^2^_p_ | |
| Main effects (Time) | 8.16 (2.31) | 8.16 (1.93) | 7.85 (2.46) | 2.921 | 0.055 | 0.02 | |
| Simple main effects | Baseline | T1 | T2 | F | p | η^2^_p_ | |
| non-experienced | 8.26 (2.19) | 8.17 (1.90) | 8.04 (2.28) | 0.524 | 0.593 | 0.00 | |
| experienced | 7.74 (2.77) | 8.09 (2.08) | 7.03 (3.02) | 1.954 | 0.150 | 0.05 | |
|  | outing | | | | | | |
|  | Baseline | T1 | T2 | F | p | η^2^_p_ | |
| Main effects (Time) | 8.61 (2.06) | 8.58 (1.89) | 8.29 (2.33) | 3.292 | 0.038 | 0.02 | |
| Simple main effects | Baseline | T1 | T2 | F | p | η^2^_p_ | |
| non-experienced | 8.64 (2.11) | 8.67 (1.84) | 8.46 (.24) | 0.631 | 0.532 | 0.00 | |
| experienced | 8.38 (1.77) | 8.00 (2.17) | 7.17(2.63) | 2.996 | 0.060 | 0.12 | |
| Upper primary students | denigration | | | | | |  |
|  | Baseline | T1 | T2 | F | p | η^2^_p_ |  |
| Main effects (Time) | 7.41 (2.33) | 7.42 (2.22) | 7.23 (2.10) | 1.246 | 0.288 | 0.00 |  |
| Simple main effects | Baseline | T1 | T2 | F | p | η^2^_p_ |  |
| non-experienced | 7.41 (2.30) | 7.61 (2.21) | 7.33 (2.12) | 1.213 | 0.299 | 0.01 |  |
| experienced | 7.42 (2.39) | 7.09 (2.23) | 7.03 (2.07) | 1.514 | 0.222 | 0.013 |  |
|  | flaming | | | | | |  |
|  | Baseline | T1 | T2 | F | p | η^2^_p_ |  |
| Main effects (Time) | 7.47 (2.50) | 7.32 (2.31) | 7.25 (2.18) | 1.069 | 0.344 | 0.00 |  |
| Simple main effects | Baseline | T1 | T2 | F | p | η^2^_p_ |  |
| non-experienced | 7.53 (2.55) | 7.49 (2.27) | 7.30 (2.23) | 0.981 | 0.376 | 0.01 |  |
| experienced | 7.33 (2.38) | 6.96 (2.38) | 7.15 (2.07) | 0.833 | 0.436 | 0.01 |  |
|  | outing | | | | | |  |
|  | Baseline | T1 | T2 | F | p | η^2^_p_ |  |
| Main effects (Time) | 7.78 (2.46) | 7.73 (2.43) | 7.38 (2.34) | 3.624 | 0.027 | 0.01 |  |
| Simple main effects | Baseline | T1 | T2 | F | p | η^2^_p_ |  |
| non-experienced | 8.00 (2.31) | 7.99 (2.33) | 7.63 (2.22) | 3.072 | 0.047 | 0.01 |  |
| experienced | 7.27 (2.74) | 7.12 (2.56) | 6.78 (2.52) | 1.158 | 0.316 | 0.01 |  |

Table 4. The effect of the programme on *engagement in cyberbullying* by lower and upper primary students, * Mean % (SD)

|  | happy slapping | | | | | | |
| --- | --- | --- | --- | --- | --- | --- | --- |
|  | Baseline* | T1 | T2 | F | p | η^2^_p_ |  |
| Main effects (Time) | 85.43 (35.35) | 86.61 (34.12) | 83.46 (37.22) | 0.696 | 0.499 | 0.00 |  |
| Simple main effects | Baseline | T1 | T2 | F | p | η^2^_p_ |  |
| lower primary students | 90.91 (28.88)0 | 93.64(24.52) | 94.55 (22.81) | 1.000 | 0.370 | 0.01 |  |
| upper primary students | 81.25 (39.17) | 81.25 (39.17) | 75.00 (43.45) | 2.270 | 0.105 | 0.02 |  |
|  | exclusion | | | | | | |
|  | Baseline | T1 | T2 | F | p | η^2^_p_ |  |
| Main effects (Time) | 77.80 (41.60) | 83.51 (37.15) | 76.32 (42.56) | 5.853 | 0.003 | 0.01 |  |
| Simple main effects | Baseline | T1 | T2 | F | p | η^2^_p_ |  |
| lower primary students | 85.63 (35.18) | 88.66 (30.54) | 83.91 (36.85) | 1.891 | 0.153 | 0.01 |  |
| upper primary students | 73.24 (44.34) | 79.93 (40.12) | 71.91 (45.02) | 5.078 | 0.007 | 0.02 |  |
|  | denigration | | | | | | |
|  | Baseline | T1 | T2 | F | p | η^2^_p_ |  |
| Main effects (Time) | 44.61 (49.77) | 47.87 (50.02) | 46.37 (49.93) | 0.480 | 0.619 | 0.00 |  |
| Simple main effects | Baseline | T1 | T2 | F | p | η^2^_p_ |  |
| lower primary students | 34.01 (47.54) | 34.69 (47.76) | 36.73 (48.37) | 0.202 | 0.817 | 0.00 |  |
| upper primary students | 50.79 (50.09) | 55.56 (49.79) | 51.98 (50.06) | 0.991 | 0.372 | 0.00 |  |
